# Bioinformatics in Mexico: A diagnostic from the academic perspective and recommendations for a public policy

**DOI:** 10.1371/journal.pone.0243531

**Published:** 2020-12-15

**Authors:** Dagoberto Armenta-Medina, Christian Díaz de León-Castañeda, Brenda Valderrama-Blanco

**Affiliations:** 1 Dirección de Cátedras, CONACYT Consejo Nacional de Ciencia y Tecnología, Ciudad de México, México; 2 INFOTEC Centro de Investigación e Innovación en Tecnologías de la Información y Comunicación, Aguascalientes, México; 3 Instituto de Biotecnología, Universidad Nacional Autónoma de México, Cuernavaca, Morelos, México; University of Bologna, ITALY

## Abstract

In this work, we present a diagnostic analysis of strengths, weaknesses, opportunities and threats (SWOT) of the current state of Bioinformatics in Mexico. We conducted semi-structured interviews among researchers and academics with key expertise in this field, identified by bibliometric analyses and qualitative sampling techniques. Additionally, an online survey was conducted reaching a higher number of respondents. Among the relevant findings of our study, the lack of specialized human resources and technological infrastructure stood out, along with deficiencies in the number and quality of academic programs, scarce public investment and a weak relationship between public and private institutions. However, there are great opportunities for developing a national Bioinformatics to support different economic sectors. In our opinion, this work could be useful to favor a comprehensive network among Mexican researchers, in order to lay the foundations of a national strategy towards a well designed public policy.

## Introduction

Bioinformatics is the application of computational technologies to manage and analyze biological data [1]. Nowadays, this data is produced by several "Omics" technologies and has been classified according to the biological molecule studied: Genomics when is obtained from nucleic acids (DNA or RNA); Proteomics when is obtained from proteins; Metabolomics when is obtained from metabolites and so on. All this information, taken together, reveals unknown aspects of biological systems. However, the integration of this information remains a challenge.

Currently, Bioinformatics requires the continuous development of two complementary technological fields: the "Omics" technologies necessary for the description or sequencing of biological molecules and the computational tools for data management. The latter is only possible thanks to the development of new tools and algorithms aiming to continuous improvements in storage and management of information, computing power, data processing and specialized software, as well as communication protocols for data transfer. It has been estimated that without these technological resources, the simple task of reading a single human genome "by eye", would require 26 years of work for a single person [2].

Thanks to the advance in these fields, Bioinformatics has developed specialized tools for the analysis of biological data such as the automatic annotation of genomes, the prediction of the structure and function of genes and proteins, the classification of species, the molecular detection of organisms such as pathogens or genetically modified (GMO), among many others [3].

In addition to its contributions to basic research such as the elucidation or understanding of the molecular mechanisms involved in biological systems, Bioinformatics is nowadays one of the primary areas for life sciences innovation and is highly relevant for developing global bio-economy [4]. For example, in the health care sector Bioinformatics has the potential to generate useful information for decision making in clinical and public health settings. It’s also a tool for biomedical research in early diagnosis and therapeutics development. In the agricultural sector, Bioinformatics is a tool for genotyping organisms for the food industry. Regarding the environmental sector, Bioinformatics is needed to characterize at a genetic level, new organisms from natural environments and can help to assess the impact of pollution on ecosystems, providing valuable information for its restoration.

Bioinformatics has shown a prominent advance in several high-income countries such as the United Kingdom, Switzerland, Germany, and the United States of America, which have generated strategies for the development of bioindustries and life sciences [4–7]. It is noteworthy the situation of some low to middle-income countries, such as India, where policies have been implemented since the early 1980s for the development of Bioinformatics with good known results [8, 9].

Latin American countries such as Brazil, Argentina, and Costa Rica have developed strategies through the consolidation of Bioinformatics networks and associations that have allowed them to gradually solve problems such as the lack of expertise, human resources and infrastructure in their countries [10, 11]. However, these countries haven’t reached a critical mass of experts on the field to cope with the current advance in “Omics”.

Mexico has a rich biodiversity and a human population with a mixed genomic background. Therefore, the burst of biological data through Genomics and its study by bioinformatics tools constitutes an opportunity to develop different life and health sciences. Bioinformatics has progressed in this country in several ways, including advances in Information and Communication Technologies (ICT) in synergy with academic programs in Genomic Sciences (Table 1). An article published in 2007 described some aspects of the state of Bioinformatics in Mexico, mentioning ongoing genomic projects, educational programs in Genomics, and other resources developed by Mexican institutions [12]. Recent work showed that in Latin America, Mexico is second place in scientific production (based on number of publications) related to Bioinformatics, only after Brazil; interestingly, it leads in collaborations within the region [13]. The impact of Bioinformatics has reached other sectors, such as Biotechnology [14, 15] and health care disciplines [16–18]. However, it is not clear if factors such as specialized training, infrastructure, financing and technological development are essential to lead the sector towards international competitiveness. Moreover, there is a lack of studies taking the academics’ voice to identify the current status and the opportunities for Bioinformatics in the country.

**Table 1:** Timelime of bioinformatics in Mexico^a^.

Year	Action
1997	Mexican researchers collaborate in the *Escherichia coli* genome sequencing project. UNAM [19]
2006	Mexican researchers publish the first large-scale sequencing project: *Rhizobium etli* genome. CIFN/CCG, UNAM [20]
2006	Mexican researchers lead the *Taenia solium* genome sequencing project and participate in other cestode genome projects. UNAM [21]
2003	The *Licenciatura en Ciencias Genómicas* [Genomic Sciences Bachelor’s Program] was approved at UNAM. Source: http://oferta.unam.mx/ciencias-genomicas.html
2004	The *Instituto Nacional de Medicina Genómica* [National Institute of Genomic Medicine] (INMEGEN) was launched within the *Institutos Nacionales de Salud* [National Institutes of Health], SSA. Source: https://www.inmegen.gob.mx/
2005	The *Laboratorio Nacional de Genómica para la Biodiversidad* [National Laboratory of Genomics for Biodiversity] (LANGEBIO), CINVESTAV, was launched. This research center focus is plant genomics. Source: https://langebio.cinvestav.mx/
2007	The *Sociedad Mexicana de Ciencias Genómicas* [Mexican Society of Genomic Sciences] was created. Source: http://smcg.ccg.unam.mx/
2007	The *Nodo Nacional de Bioinformática* (*Nodo Mexicano EMBNet*) [National Bioinformatics Node] was created, UNAM. Source: http://congresos.nnb.unam.mx/TIB2019/
2015	The *Red de Apoyo a la Investigación* (RAI), *Unidad de bioinformática*, *bioestadística y biología computacional* [Reasearch Support Network, Bioinformatics, Biostatistics and Computational Biology Unit] was created. Consortium UNAM and *Institutos Nacionales de Salud* [National Institutes of Health], SSA. Source: http://rai.unam.mx/
2012	The *Laboratorio Nacional de Cómputo de Alto Desempeño* [National High Performance Computing Laboratory] (LANCAD) was launched. Consortium CIVESTAV, UAM and UNAM. Source: http://www.lancad.mx/
2015	The *Centro de Ciencias de la Complejidad* [Complexity Sciences Center], UNAM, was launched. This center incorporates research about genomic regulation systems and networks. Source: https://www.c3.unam.mx/
2019	The *Red Mexicana de Bioinformática* [Mexican Bioinformatics Network] was created. Source: https://www.redmexicanadebioinformatica.org/

^a^Acronyms:

CCG: Centro de Ciencias Genómicas (UNAM); CINVESTAV: Centro de Investigación y de Estudios Avanzados; LANGEBIO: Laboratorio Nacional de Genómica para la Biodiversidad; SSA: Secretaría de Salud; UNAM: Universidad Nacional Autónoma de México; UAM: Universidad Autónoma Metropolitana; INMEGEN: Instituto Nacional de Medicina Genómica (SSA).

This study proposes an analysis of the strengths, weaknesses, opportunities, and threats (SWOT) of Bioinformatics in Mexico from an academic perspective. We seek to assess the current state of this biological information technology to inform the stakeholders towards the formulation of a specific public policy.

## Materials and methods

### Study design

A case study design was proposed to explore the development of Bioinformatics in Mexico from a comprehensive perspective [22]. The general approach of the study was mixed, concurrent and with qualitative predominance [23]. The qualitative component used phenomenology as the principal theoretical approach [24], which is based on the exploration of personal perceptions through the experience with the phenomenon under study.

### Data collection

Semi-structured interviews were conducted among researchers working in Bioinformatics in Mexico. A preliminary interview guide was prepared that included items to explore the strengths, weaknesses, opportunities, and threats (SWOT) related to the development of Bioinformatics in Mexico. This analysis has been applied successfully in the study of organizations, countries, groups, and individuals on specific issues, identifying problems, and proposing strategies in different fields [9, 25].

Given the nature of the study, the interview guide was adapted during the research to explore emerging subtopics. Additionally, a structured online survey was carried out through Google Forms in order to explore some additional topics related to the main topic of interest. Within these topics, the status and opportunities of Bioinformatics were explored in different economic sectors (health care, pharmaceutics, agriculture and the environment).

### Recruitment of respondents

To identify the leading researchers carrying out Bioinformatics research in Mexico, a bibliometric analysis was carried out using the PubMed database [26]. For this search, all the articles published between 2008 and 2018 with the authorship or co-authorship of at least one researcher affiliated to a Mexican institution were taken into account. The words equivalent to "bioinformatics" and "computational biology" were used as search criteria. In the first instance, the analysis identified top researchers by their outstanding number of publications as key respondents in the field.

Additionally, qualitative sampling techniques such as purposeful, convenience, opportunity and snowball were used to include more key respondents [27]. The diversity within respondents based on the work role (researcher, services laboratory chief, laboratory analyst, enterprise manager); type of affiliation (whether public or private institutions); and the economic sector(s) in which they work (i.e., health care, agriculture, or environment). The main inclusion criterium was being a researcher or academic in Bioinformatics affiliated to a Mexican institution related to life sciences. Other respondents from foreign institutions were also included to expand the view of Bioinformatics, as long as they had studied, worked, or collaborated in this field in Mexico.

Interviews with selected respondents were audio-recorded with verbal consent. Previously, the participants were informed about the objective of the study, confidential handling of the information, as well as their rights based on ethical criteria during social research. Each respondent was assigned a numerical tracking code for confidentiality.

### Data analysis

Audio-recorded interviews were transcribed and analyzed using techniques of qualitative content analysis [28] through a Google Sheets shared file. Emergent codes were identified and cataloged within the four major categories of SWOT analysis (Strengths, Weaknesses, Opportunities and Threats). The information obtained from the online survey was analyzed through descriptive statistics.

### Other ethical considerations

The protocol was reviewed by an academic board of the *Centro de Investigación e Innovación en Tecnologías de Información y Comunicación* (INFOTEC) (Approval number: INFOTEC-DAIC-2S.21-20180101BIOINFOTEC). Researchers that conducted the study are trained and have previous experience in ethics of qualitative research including humans.

## Results

### Bibliometric analysis and respondents sampling

**Fig 1:**
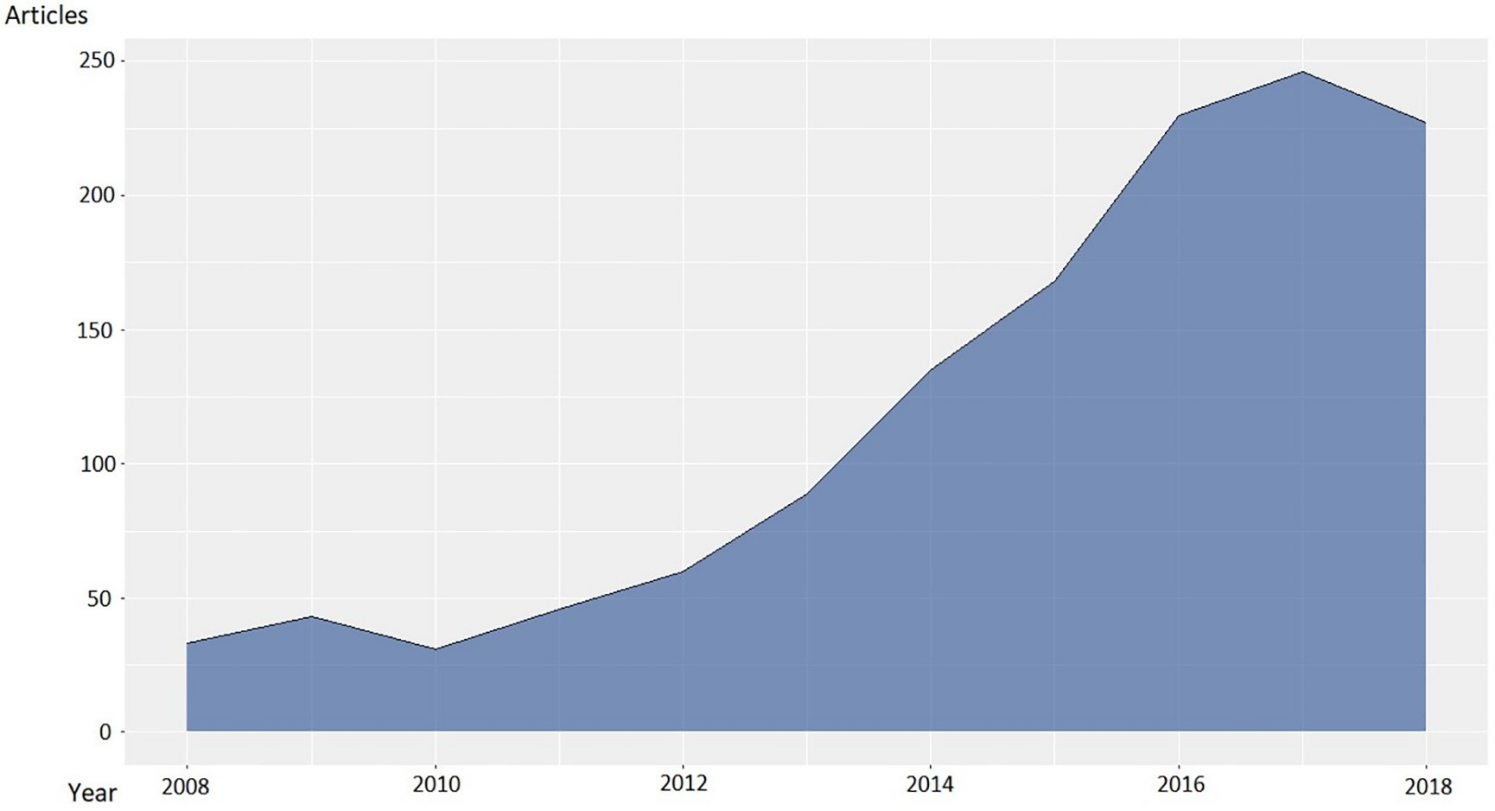
Dynamics of publications related to Mexican bioinformatics between 2008 and 2018 (PubMed database).

Through the bibliometric analysis, it was observed that publications in the area of Mexican bioinformatics grew considerably since 2012 (Fig 1). This analysis helped to identify the principal researchers in the field of Bioinformatics in Mexico, based on their number of publications.

### Individual perspectives from researchers and academics

A total of 26 researchers were selected for both qualitative and quantitative approaches; from them, 14 researchers were approached through semi-structured interviews, 12 researchers were surveyed through the online questionnaire, and five with both. Table 2 shows a summary of the general profile and the research techniques applied for each selected respondent.

**Table 2:** Respondents selection and profile^a^.

Respondent number	Sampling technique	Sex	Institution of affiliation	Institution type	Work role	Main economic sectors	Semi-structured interview	Online survey
1	B	M	LANGEBIO-CINVESTAV	N, public	R	M	✓	✓
2	B	M	IBT-UNAM	N, public	R, LC	M	✓	
3	B	M	*Wilfrid Laurier University*	F, public	R	M	✓	
4	B	M	INMEGEN-SSA	N, public	LC	H	✓	
5	C/O	M	INSP-SSA	N, public	R	H	✓	
6	C/O	M	*Cambridge Precision Medicine*	F, private	EM	H	✓	
7	C/O	M	ENES-UNAM	N, public	R	M	✓	
8	B	M	TEC-Mty	N, private	R	H	✓	✓
9	B	F	IIMAS-UNAM	N, public	R	H	✓	
10	P	M	LANGEBIO-CINVESTAV	N, public	LC	A	✓	✓
11	P	F	INMEGEN-UNAM	N, public	LA	H	✓	✓
12	B	M	IBT-UNAM	N, public	R, LC	M	✓	
13	P	F	UAEM	N, public	R	H	✓	✓
14	C/O	M	ENES-UNAM	N, public	R	M	✓	
15	P	F	LANGEBIO-CINVESTAV	N, public	R	H, A		✓
16	P	M	CIIDIR-IPN	N, public	R	A		✓
17	P	M	CCG-UNAM	N, public	LC	M		✓
18	B	M	CINVESTAV Irapuato	N, public	R, LC	M		✓
19	B	M	ENCB-IPN	N, public	R	M		✓
20	P	M	CCG-UNAM	N, public	R	M		✓
21	P	M	CNyN-UNAM	N, public	R	M		✓
22	B	M	ESM-IPN	N, public	R	H		✓
23	P	M	CICESE-CONACYT	N, public	R	H		✓
24	P	F	*Código 46*	N, private	EM	H		✓
25	P	M	INECOL-UNAM	N, public	R	M		✓
26	P	M	IBT-UNAM	N, public	R	M		✓

^a^Acronyms:

**Sampling technique used for respondents selection**: B: Bibliometric analysis; P: Purposeful sampling; C/O:Convenience/Opportunity sampling; SB: Snowball sampling.

**Sex**: M: Male; F: Female

**Institution of affiliation**:

**Public Institutions**: CCG: Centro de Ciencias Genómicas; CIIDIR: Centro Interdisciplinario de Investigación para el Desarrollo Integral Regional; CINVESTAV: Centro de Investigación y de Estudios Avanzados; CISESE: Centro de Investigación Científica y de Educación Superior de Ensenada; CNyN: Centro de Nanociencias y Nanotecnología; CONACYT: Consejo Nacional de Ciencia y Tecnología; ENCB: Escuela Nacional de Ciencias Biológicas; ENES: Escuela Nacional de Estudios Superiores; ESM: Escuela Superior de Medicina; IBT: Instituto de Biotecnología; IIMAS: Instituto de Investigaciones en Matemáticas Aplicadas y en Sistemas; INMEGEN: Instituto Nacional de Medicina Genómica; INECOL: Instituto de Ecología; INSP: Instituto Nacional de Salud Pública; IPN: Instituto Politécnico Nacional; LANGEBIO: Laboratorio Nacional de Genómica para la Biodiversidad; SSA: Secretaría de Salud; UAEM: Universidad Autónoma del Estado de Morelos; UNAM: Universidad Nacional Autónoma de México.

**Private Institutions**: TEC-Mty: Instituto Tecnológico de Monterrey.

**Institution type**: N: National; F: Foreign.

**Work role of respondent**: R: Researcher; LC: Research/Services Laboratory Chief; LA: Laboratory Analyst; EM: Enterprise manager.

**Main economic sectors where respondents work**: H: Health care; A: Agricultural; E: Environment; M: Multisector.

#### Strengths

*Competitive academic and research levels in life sciences and ICT*. The respondents considered that the areas of biological sciences and computing in Mexico have consolidated on their own and may converge together to strengthen Bioinformatics. It is perceived that there is an excellent potential for the development of Bioinformatics, given the good level of professionals from the main domains where it emerges. However, adequate dialogue is still to be established.

"I think that there is much workforce from the computational point of view and this workforce is very consolidated, the biology part is a very consolidated sector too, so the only thing that is missing, I think, is to put aside that silence between them and make those two sectors communicate between them"

(Respondent 14).

*Diversity among bioinformatics research interests*. Although the number of researchers and other human resources in Bioinformatics is small in Mexico, there are currently several groups or individual efforts in different areas aiming at diverse economic sectors. Research interests span from traditional topics such as evolution, regulation of gene expression, structural and functional genomics, to advanced topics such as systems biology and applied biodiversity studies. In this sense, Mexico is perceived as one of the countries with the most significant advances in Bioinformatics in Latin America.

"From Latin America, we are one of the countries that have had more progress, possibly because of the proximity to the United States, and the exchange of researchers"

(Respondent 2).

**Fig 2:**
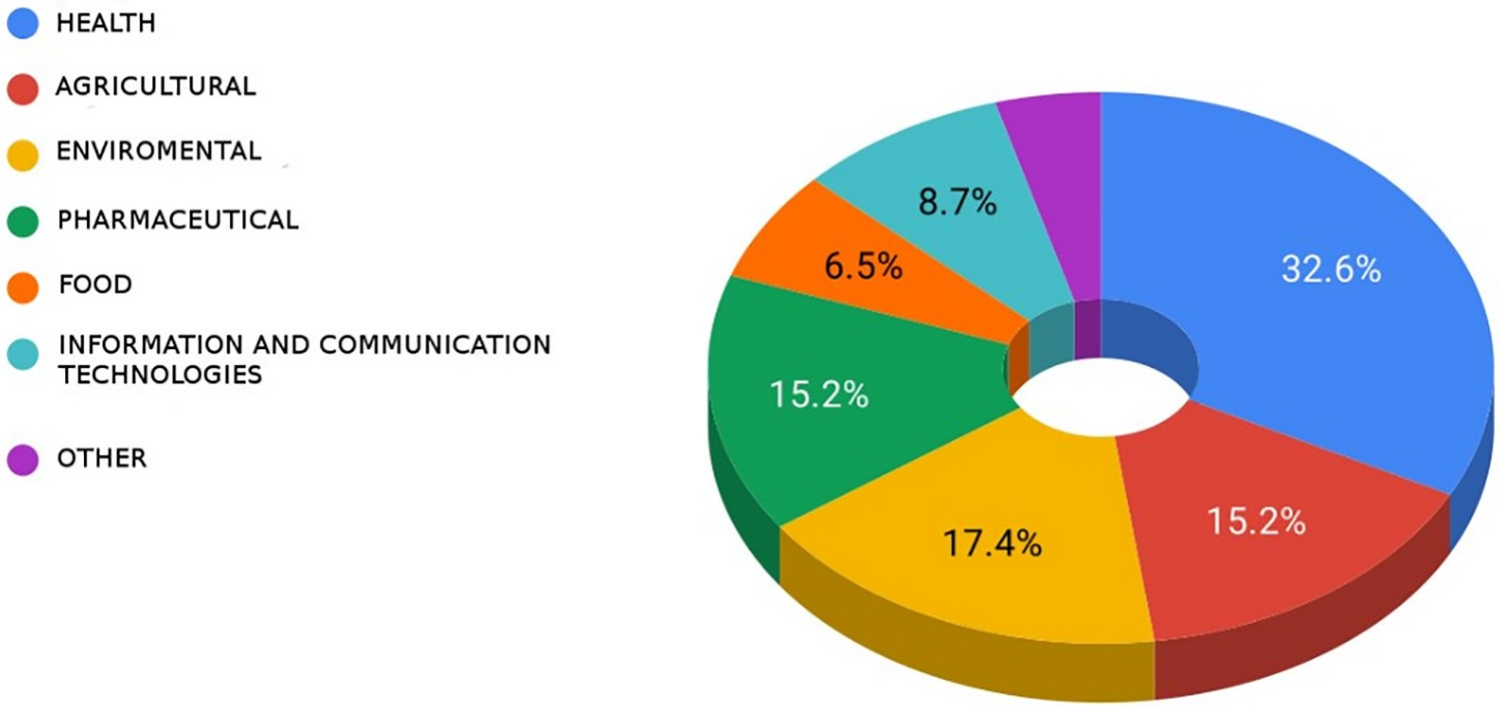
Respondents’ research in particular economic sectors through bioinformatics.

Surveyed respondents reported that their Bioinformatics research is in the health (32.6%), environmental (17.4%), agricultural (15.2%), and pharmaceutical (15.2%) sectors (Fig 2).

#### Weaknesses

*Lack of human resources and educational programs in bioinformatics*. One of the more significant coincidences among interviewees was the need for human resources in Bioinformatics and, consequently, the weak supply of academic programs in the field.

"I think right now, the bottleneck is not the quality of the human resource but the number of people, but that is a general evil, worldwide there is a need of bioinformaticians and Mexico is not the exception…"

(Respondent 12).

"It would be a matter of more personal training or to send people who are involved in bioinformatics to be trained abroad"

(Respondent 10).

"I think it is a little disjointed, that there is not yet an institutional effort regardless of the institution, that allows to carry out a structured academic training, from my perspective a lot of the bioinformaticians in the country have learned the job through small and separate courses that are offered, not from a structured syllabus"

(Respondent 7).

*Lack of infrastructure and capacity for the management of computer technology resources*. Many of the interviewees mentioned the lack of different types of infrastructure necessary for information storage and processing, as well as low speed and poor connectivity of the networks.

"More supercomputer nodes are needed, I believe, in such a way that they are close and that there is no need to send information far away… and better connectivity… "

(Respondent 4).

"There is a need to invest in these important hardware components because it limits Mexico’s development as possible bioinformatic power. A good starting point is to have an infrastructure of storage and processing servers, for we as bioinformaticians to do the work that is needed"

(Respondent 10).

Regarding connectivity, the following was commented:

"For those of us who do data analysis, our data entry sets are extensive, that is, terabytes… sometimes petabytes of data… then transfer that data over an internet network that is not very high speed it is complicated"

(Respondent 4).

"We always depend on connectivity in Mexico, although we have been improving little by little, the speed of connection of Mexican research centers has been ridiculously below the research centers abroad"

(Respondent 1).

*Lack of communication between researchers in the field of bioinformatics*. One of the themes that the interviewees repeatedly pointed out was the lack of integration of bioinformaticians in the country, noting that national bioinformatics occurs through very isolated efforts. In this regard, the interviewees mentioned the following,

"I believe that one of the things that we have also lacked as a community of researchers in this area is to unite a little strength, right? There are many things around computational biology in which we would communicate more if there were more networking between researchers and between institutions"

(Respondent 4).

In general, the respondents agreed that it is crucial to look for mechanisms to join efforts in the key areas of Bioinformatics, in such a way that it may result in more successful complementation of resources.

*Limited availability of sequencing data from Mexican species and human populations*. There are currently many approved genomic and metagenomic sequencing projects endorsed with public financing, which have generated a large amount of data. However, its interpretation continues to represent a great challenge. On the other hand, it should be noted that broad coverage in the sequencing of Mexican species is still pending. Also, it is identified that when biological data is available, it is not interrelated and dispersed.

"Mexico is a focus of biological diversity, so I do not know why we are not doing that, sequencing genomes in a systematic way, we have a lot of important endemic species, and I think we have to do that work"

(Respondent 8).

Likewise, we identified the need for specialized databases in Bioinformatics to concentrate the information of Mexican species and human populations.

"There are many databases that suffer from maintenance; there is not a development plan beyond the researcher who created it"

(Respondent 4)

"We do not have, for example, an ‘NIH’ or an ‘NCBI’ [referring to the *National Center for Biotechnology Information*, an international public database for biological data made available by the *National Library of Medicine* (NLM) that is supported by the *National Institutes of Health* (NIH)]; we do not have a center that concentrates Mexican biological data, a center in charge of putting all that available… because the truth is that the researchers or how this has been done are due to the efforts of individual groups making the development very slow and fragmented"

(Respondent 8).

"What I think is necessary, is to make a national repository, where you can have an impulse to put or upload information that is available to the Mexican community that is related to ‘x and y’ topic, in addition to putting it parallel in the international database. The problem is that each one [each researcher] is making an independent effort and is not a concentrated effort"

(Respondent 14).

*Lack of regulatory frameworks on biological data*. The regulatory framework for the management of biological data in Mexico is weak, hindering Bioinformatics development. At present, many of the biological data used for bioinformatic analyses (genomic, genetic or clinical) are of sensible use, especially those from human sources, rising privacy problems, and ethical dilemmas.

"In Mexico and other parts of the world, the regulatory part is still absent. Of course, on the one hand, our legislators, or those who could make legal initiatives, are not familiar or in direct contact with the area, so the initiative must also come from the scientist, we should be involved and participate in this regulation, that is basic"

(Respondent 12).

"The other thing has to do with a question of legislation, the matter of data protection which is, of course, fundamental in the case of patients. Often it is so complicated to have access to clinical files"

(Respondent 4).

"In fact, it is a very important bottleneck, because signed consents have to include where the information is going to be and what use will be given to this information, and I think that any system that is integrated into the network is vulnerable, so, I do not think that a country like ours is in a position to have sufficiently secure and confidential databases…"

(Respondent 11).

*Structural delays in bioinformatics applications or prototypes developments*. Derived from the late adoption of complementary technologies and mainly due to reduced human resources and projects focused on bioinformatics, the development of applications or prototypes in this area is very scarce in the country, if not null. Also, the respondents mentioned the need to promote these applications or prototypes, especially in high impact sectors such as health care.

"I work specifically in cancer research, I read papers and I see these ideas and huge gaps, and I think cancer is the most studied topic, I can’t imagine things that are not studied so extensively. For example, at this symposium, I saw many studies where almost all of the things that are proposed are ‘artisanal’ in the sense that they don’t use a software"

(Respondent 8).

*Excessive bureaucracy in the processes for technical services into the academy*. The bureaucratic and normative barriers common in Mexican public institutions are a threat to the development of Bioinformatics in Mexico. There are slow and intricated procedures for the usage of bioinformatics resources such as consultancy, software and rent of computational infrastructure.

"The bureaucracy does not allow you to use the resources that you think convenient, for example, in Canada until recently was forbidden to buy cloud services with your grants [project grants or financial supports], even though it could be cheaper than buying a computer. That is changing, but I think it is important that the bureaucracy does not interfere…"

(Respondent 3).

"We have some projects in which we do analysis outside the country in collaboration with the National Supercomputing Center in Barcelona. The situation is that data is sensitive and I am not allowed to send it to other countries, because of legal restriction issues"

(Respondent 4).

#### Opportunities

*Access to international public databases for bioinformatics analysis*. The development of Bioinformatics, national- or international-wide, benefits from the availability of public databases. Large amounts of biological and genomic data improve our understanding and predictability of biological phenomena through bioinformatics applications. In turn, it contributes to the development of new computational tools. For example, the availability of data for machine learning training processes.

"But what I have seen, every time someone finds a new gene, along with sending the article to the journal, they send the sequence to NCBI. Then, we have the advantage that the repository is already there"

(Respondent 13).

*Reduction of sequencing costs*. Respondents identified that, within the international context, many of the technologies associated with Bioinformatics, such as "Omics" and ICT, are highly developed and commercially competitive, reducing costs. This context is an opportunity for Mexico, facilitating the generation of new bioinformatic products from simple applications to more elaborate services.

"Sequencing becomes cheaper; we have more computing capacity. In fact, already the cost of sequencing goes down much faster than Moore’s Law"

(Respondent 4).

"The advantage of bioinformatics is that you do not need to do all the experiments yourself, and as the sequencing is getting cheaper and cheaper, you can send your samples for sequencing to China and analyze them in Mexico. I think this is one of those areas where Mexico can be very competitive because you do not need to have the whole laboratory, you do not need to have everything from the beginning to do it. Now you can do much outsourcing; you can send it to sequence on one side of the world, use the cloud services to do the computational part, and we have brilliant people in computing and technology"

(Respondent 3).

"Maturity of the fields, that is, before I despaired for having the last bacterial genome in the database because there were very few 15, 10, 20, and now there are about 200 thousand. Now it is trivial to have genomic sequences, this facility with which sequences are generated indicate that it is time to make applications"

(Respondent 3).

*Availability of free software for bioinformatics*. The availability of free software favors the development of bioinformatics in the academic environment by avoiding redundancy and reducing costs.

"The work teams that I manage are not big enough to have a section that does software development and another that uses it, so I declare myself a user, and fortunately, there is excellent and free software"

(Respondent 13).

*Biological diversity in the country*. Mexico is one of the countries with the highest biodiversity. It is possible through the use of Bioinformatics to explore that diversity aiming to the study of fundamental biological phenomena as well as the search for therapeutic or other commercial interest molecules.

"Speaking in populations terms, we have a lot to exploit, but as I say, we are still in the process of answering the basic questions applying these tools, which I would suggest today a potential market closely related to what the population is seeing a lot, what they are to characterize and make personalized genomic medicine"

(Respondent 10).

*A great interest of the Mexican scientific community in the area of bioinformatics*. Researchers in the biological and health science fields show interest in the area of Bioinformatics since they are aware of the data burst in the field. There is an intention above all of the people in these areas to exploit Bioinformatics. However, as described below, there are barriers to educational training that do not allow us to adapt it to the research quickly.

"People want to do it, they want to do more bioinformatics, they want to get into that world. They have to, because right now is a time of data and if you do not know how to analyze and interpret data, you are almost out of competition"

(Respondent 8).

#### Threats

*Limitation of public and private financing towards bioinformatics projects*. The interviewees referred to the low availability of funds for bioinformatics projects, especially those that involve genomic sequencing, probably because of its higher costs.

"Then it is the biggest limitation; my collaborators and I, often have ideas that seem very good to carry out for a project, but we simply do not have the money"

(Respondent 9).

"The lack of funds…, the lack of opportunities to attend large projects, requiring, for example, the interaction of bioinformatics"

(Respondent 12).

**Fig 3:**
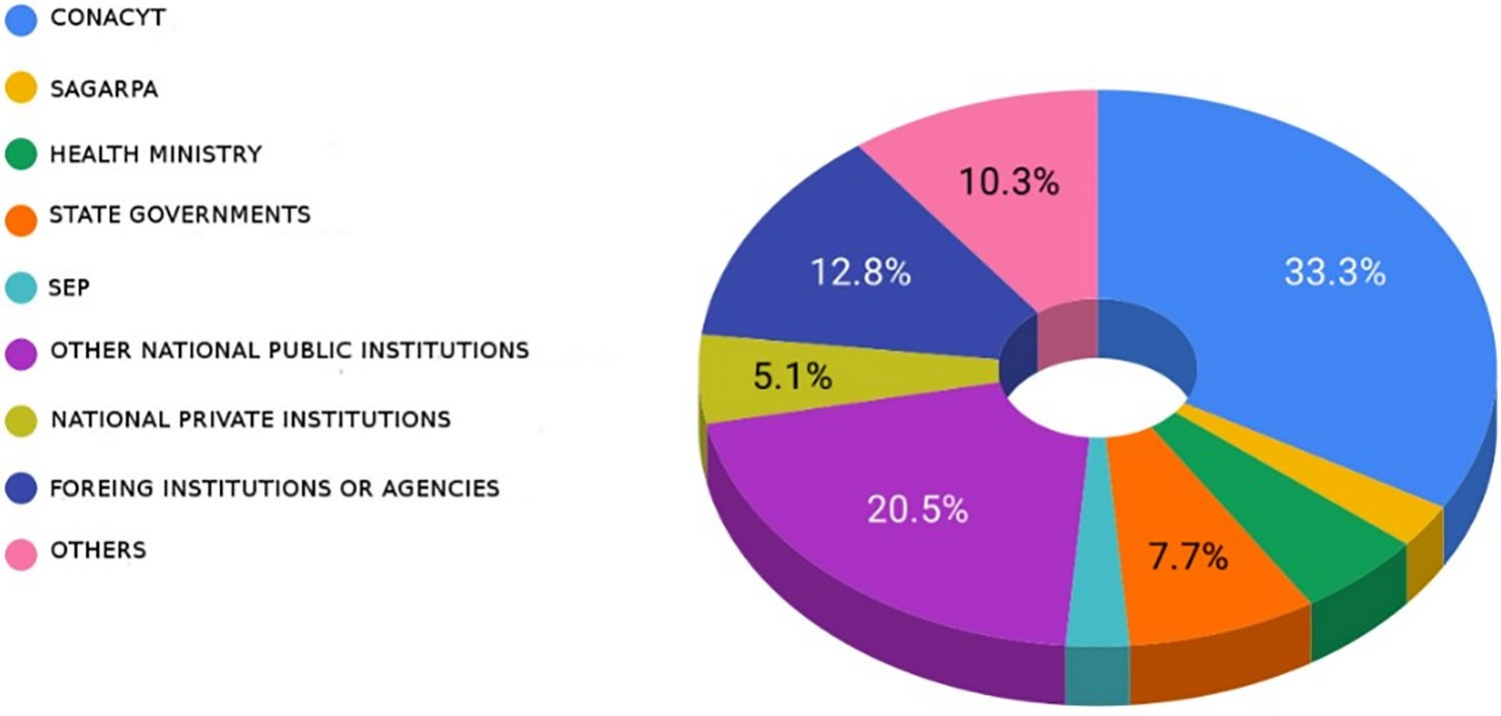
Bioinformatics projects financing sources as stated by respondents.

Through the survey, it was found that most of the funding assigned to Bioinformatics projects were public and came from the *Consejo Nacional de Ciencia y Tecnología* [National Council of Science and Technology] (CONACYT) (33.3%), with a small contribution from the private sector (5.1%) (Fig 3).

*Academic and business Mexican culture*. Respondents identified that Mexican entrepreneurs do not have the culture of investing in bioinformatics research. On the other hand, they also identified that academics are not used to pay for bioinformatics services or consultancies to support their research, but they rather prefer graduate students to tackle the problem even if it is less effective. Besides, other respondents perceive poor knowledge about the potential of Bioinformatics.

"Another issue is that Mexico always has this distrust, or people do not like to think that they have to pay money for training or analysis"

(Respondent 1).

"Sometimes people who are not well informed on these topics can delay the development of bioinformatics, minds that they think that bioinformatics is not a valuable tool and that the investment in computer equipment or personal training do not worth"

(Respondent 10).

*The exploitation of biological data from Mexican biodiversity in the hands of foreign entities*. Some respondents identified that the study of endemic species or with broad national representation has advanced in other countries mainly, leading to the exploitation of patents by foreign companies and potentially inhibiting the local use of national resources.

"In many other countries, they are moving to get information from the population of patients. There are biobanks and a huge amount of data, for example, in England, they have a very advanced project, one of 100,000 completely sequenced genomes; in whole Europe, there are many studies in GWAS [Genome Wide Associations Studies]; and Brazil has already inserted itself in that dynamics. We are lagging”.

(Respondent 8).

*Foreign competition to provide bioinformatics services*, *applications or prototype development*. It is identified that nowadays, other countries have accumulated experience to offer bioinformatics services, representing a competitive disadvantage for Mexican companies. It is worth mentioning that in many of these countries, the bioinformatics services evolution was favored by innovation and development of complementary technologies such as massive nucleic acid sequencing.

"…but precisely, as there are tasks [bioinformatics services] that can be provided from a remote distance, some countries like India, China, Korea, United States, and others have already started a series of small or large companies dedicated to providing these bioinformatics services, then it is not clear to me now what are the chances if we compete [taking about the chances for Mexico to compete in this international market]"

(Respondent 4).

"Again, the problem is that there are several companies of different sizes some quite large in the private initiative, which has been playing with those ideas of how to exploit the niche of personalized medicine and the information of genomes of millions of individuals associated with diseases and propensity to drugs, then again it is a situation that any person any country that thinks about exploring these opportunities has to be aware now, there is already a lag of about 10–15 years"

(Respondent 1).

Finally, a description of the SWOT analysis findings is presented below, and Table 3 shows a summary of these findings, as well as a ranking of importance based on the author’s criteria.

**Table 3:** SWOT analysis summary.

SWOT	Rank	Item
**Strengths**	1	Competitive academic and research levels in life sciences and ICT.
	2	Diversity among Bioinformatics research interests.
**Weaknesses**	1	Lack of human resources and educational programs in Bioinformatics.
	2	Lack of infrastructure and capacity for the management of computer technology resources.
	3	Lack of communication between researchers in the field of Bioinformatics.
	4	Limited availability of sequencing data from Mexican species or human populations.
	5	Lack of regulatory frameworks on biological data.
	6	Structural delays in Bioinformatics applications or prototypes developments.
	7	Excessive bureaucracy in the processes for technical services into the academy.
**Opportunities**	1	Access to international public databases for Bioinformatics analysis.
	2	Reduction of sequencing costs.
	3	Availability of free software for Bioinformatics.
	4	Biological diversity in the country.
	5	A great interest of the Mexican scientific community in the area of Bioinformatics.
**Threats**	1	Limitation of public and private financing towards Bioinformatic projects.
	2	The exploitation of biological data from Mexican biodiversity in the hands of foreign entities.
	3	Foreign competition to provide Bioinformatic services, applications or prototype development.
	4	Academic and business Mexican culture.

## Discussion

Based on the used methodology, we were able to reveal a diagnostic of Bioinformatics in Mexico. Among the main findings of our approach, we detected poor availability of teaching programs and contents in Bioinformatics, problems in the infrastructure and administration of bioinformatics resources, absence of an inclusive collaboration network and weakness of the regulatory framework for the biological data. Derived from our diagnostic and the lessons that can be learned from other countries, we discuss the following recommendations:

### Build a plan to improve education in bioinformatics

Despite Bioinformatics in Mexico has a presence of researchers in its different areas, the amount of specialized human resources is limited. This phenomenon seems to be international since it has been identified that Bioinformatics training has been lagging during the last 20 years [29]. Therefore, the timely implementation of a bioinformatics educational strategy can be an opportunity to raise national experts.

In particular, Bioinformatics study programs and special training courses offered in Mexico present deficiencies in content and coverage in the country. Most of the respondents agreed that there is a need for a comprehensive strategy to improve Bioinformatics training. The most recurrent proposals regarded the creation of academic programs at the undergraduate and postgraduate levels. Besides, it was suggested that Bioinformatics should be incorporated in the early stages of life sciences and computing undergraduate programs in order to promote confidence and skills in students, as has been done in other countries [30].

Although there is currently a National Node of Bioinformatics constituted by some Mexican public Universities, it is not yet widely known. The node has promoted training courses in Bioinformatics along with international agencies such as the European Bioinformatics Institute (EBI) and CABANA, which is a capacity strengthening project for bioinformatics in Latin America. However, these efforts, although laudable, have been intermittent and scarce for the needs of human resources in Bioinformatics in the country.

It might result necessary to create short programs in Bioinformatics for recently graduated professionals in other areas. By doing this, professionals who are at an advanced stage of their careers could be trained by means of short workshops and online courses. Training in Bioinformatics for professionals who graduated in areas such as health care and agriculture has been successfully performed through visits from international researchers and guest trainers in some African countries [31]. These courses have been complemented by academic exchange programs with international peers [32]. Another international experience in these modalities is Australia, where a search for training material was implemented to improve pedagogy in Bioinformatics [33].

It is also necessary to train teachers to obtain the best teaching practices and keep them updated. Teacher’s training could be achieved through the temporary hiring of visiting professors from other countries as well as the participation of Mexican researchers in academic or post-doctoral training programs abroad. In this sense, one of the advantages of Mexico for the training of academics is the existence of bilateral agreements with the United States of America, such as the UC-MEXUS and COMEXUS programs that promote the exchange of researchers from various areas, favoring academic collaboration and training [34]. Also, there have been special agreements between CONACYT and particular foreign universities (i.e., Harvard University) that promote MSc and Ph.D. studies for Mexican students.

Regarding the need in Mexico to create special postgraduate programs, there are some examples to be followed. Brazil created a multi-center postgraduate program by the Association for Bioinformatics and Computational Biology (AB3C) and the University of Minas, in such a way that knowledge has been transmitted from regions with considerable research experience to states with a greater need to acquire it [35]. The implementation of this multi-center graduate program is partially responsible for the high number of publications of Brazil in Bioinformatics [10]. In addition, Central American countries such as Costa Rica and Guatemala have also developed graduate degrees in Bioinformatics through collaboration between researchers from various countries in the region [11].

### Strengthen collaborative networks in bioinformatics

In the first world and developing countries such as India, collaborative networks have favored the development of Bioinformatics, favoring the support of bioindustries [36–38]. The creation of bioinformatics networks is a significant advance to exchange experience and promote collaborative work, boosting developed countries such as the German Network for Bioinformatics Infrastructure (deNBI) case [33]. Also, in Australia, during February 2016, a national network of bioinformatics infrastructure services was relaunched: EMBL Australia Bioinformatics Resource (EMBL-ABR) which has a coordination center hosted at the University of Melbourne and ten nodes in Australia dispersed in institutions of tradition in the country, each working in critical areas [33]. In the African region, the H3ABioNet network provides bioinformatics support for the Human Heredity and Health in Africa (H3Africa) initiative focusing on the construction of infrastructure and implementation of tools that allow collaborations and data transfer through limited resources on the continent [31].

Some Latin American countries have also obtained excellent results through the use of collaborative networks for Bioinformatics development. In 2006, ten Bioinformatics research networks were formed in Brazil, facilitating the development of agricultural and livestock areas [35]. At the Central America region, the Central American Bioinformatics Network (BioCANET) focuses on providing computer support for molecular biology, as well as a service platform for the analysis of applied biomedical data. The National Bioinformatics subnet of Guatemala RedBioNaGual was created between 2013 and 2014 derived from the BioCANET network; during these years, the network programmed 14 workshops, promoting literacy in Bioinformatics [11].

Considering that Mexico presents a critical mass and quality of ICT and biological sciences professionals, we believe that the focus towards the creation of a collaborative and coordinated network of researchers and academics will be critical to implement the strategies outlined here. We consider that this vision is essential to develop collective strategies and to coordinate efforts for the exchange of technological infrastructure, courses organization, and other educational strategies, as means of enabling an organized response front to tackle emergent problems (i.e., environmental or epidemiological).

It is possible to endorse collaborative efforts in Mexico, as demonstrated by the inter-institutional work created in response to the Covid-19 pandemic. Thanks to this collaboration, several locally isolated Coronavirus strains have been sequenced since the start of the pandemic by the *Instituto de Diagnóstico y Referencia Epidemiológicos* [Epidemiological Diagnostics and Reference Institute] (INDRE, *Secretaría de Salud*), the *Instituto de Biotecnología* [Biotechnology Institute] at UNAM and some National Health Institutes.

It is important to mention that during the development of this study, a Mexican bioinformatics network was generated (*Red Mexicana de Bioinformática*), which could serve as a basis for the development of the strategies outlined here. However, there is scarce information about the members, their organization and dynamics, and the processes to have a voice within this network.

A comprehensive way to form an extensive collaborative network in Bioinformatics can be achieved by calling to a national summons. This summons would result in a record of the country’s Bioinformatics infrastructure and areas of expertise, which will serve to generate a public directory where people interested in the area can join for a common or complementary interest, enhancing interaction. This national network will favor the interaction between research nuclei, national infrastructure of information technologies such as supercomputing units, managing to coordinate existing efforts, add value, generation of training and greater dissemination of Bioinformatics in the community. Parallel to the registry, a repository of information on Bioinformatics resources can be made with Mexican participation, as teaching material derived from national projects and free software generated in the country. In the end, this network would be vital in disseminating information to the Bioinformatics community and to the general public, informing them of calls and important events.

### Build a plan to improve the ICT infrastructure and management

Our analysis identified that there are different weaknesses regarding the technological infrastructure necessary for Bioinformatic processes. Despite the existence of several supercomputing facilities in academic institutions (such as *Universidad Nacional Autónoma de México* [National Autonomous University of Mexico] (UNAM); *Centro Nacional de Supercómputo* [National Supercomputing Center]; *Universidad Autónoma Metropolitana* [Metropolitan Autonomous University] (UAM); *Centro de Investigación Científica y de Educación Superior de Ensenada* [Center for Scientific Research and Higher Education of Ensenada] (CICESE); and *Centro de Investigación y de Estudios Avanzados* [Center for Research and Advanced Studies] (CINVESTAV)), there is need for a nationwide strategy to coordinate these resources more efficiently. These institutions have appointed researchers to manage this infrastructure in addition to their research, generating work overloads. A recommendation based on the management of supercomputing resources in other countries is that the administration should be carried out by people who dedicate their time exclusively to this task and who have an inter-institutional accreditation, as well as direct communication with the diverse communities of users to initiate feedback. Well-defined protocols should accompany the administration on the provision of national supercomputing resources, such as access to users, installation of software of interest and training for their use. Another option for improving the management of supercomputing services is the payment of online services that can facilitate access to resources, although it is worth mentioning that the protocols to pay for this type of service with public funding is not sufficiently clear.

Software infrastructure is considered among respondents as a favorable aspect for Bioinformatics in Mexico. Thanks to the fact that there is an active utilization of free software, basic software applications are available, although there is a deficiency in the creation of more specialized software with different application approaches.

Concerning storage resources and biological data management, respondents identified that the scientific community lacks a national information system or repository that coordinates efforts in a similar way to the base EBI in Europe, NCBI in the United States or like those in developing countries, like the Institute of Bioinformatics of India [9, 39, 40]. In Mexico, high-quality research is being currently carried out exploring Mexican biodiversity, such as the metagenome project in the Gulf of Mexico (CIGOM) [41] and in Cuatro Ciénegas [42], or other genomic studies of the Mexican population (including indigenous population) aimed to develop genomic medicine and pharmacogenomics [43–45]. Due to the generation of this mass of information, a national repository would facilitate access to Mexican biodiversity for the solution of national problems in sectors such as health and environment through bioinformatic studies.

It should be noted that the generation of a national repository would not start from scratch. Currently, the *Comisión Nacional para el Conocimiento y Uso de la Biodiversidad* [National Commission for the Knowledge and Use of Biodiversity] (CONABIO) operates the *Sistema Nacional de Información en Biodiversidad* [*National System of Information on Biodiversity*] (SNIB), integrating information about six million records of specimens and biological observations mainly from zoological and herbal collections. This database could be further updated with information derived from genomic projects incorporating approaches such as massive sequencing of nucleic acids, microarrays, ESTs (expressed sequence tags), SNPs (single nucleotide polymorphisms) among others, creating a catalog with greater visibility to the public and private scientific community. Management of these databases can be improved by taking into consideration the needs of the Bioinformatics community under the supervision of panels of experts and bioethics committees.

Another relevant aspect in which most of the respondents agreed is the limiting speed of internet services indicating the need for an improvement in the network infrastructure throughout the country. The connectivity was temporarily relieved by the *Red Nacional de Impulso a la Banda Ancha* [National Network to Promote Broadband] (NIBA) network, which provided connectivity services to public Educational and Health Centers, as well as Government Offices, using the capacity that is available in the fiber-optic infrastructure of the *Comisión Federal de Electricidad* [Federal Electricity Commission] (CFE). Nevertheless, connectivity has not been sufficiently improved to achieve an agile handling of massive data. In this regard, a recommendation is to generate a proposal from the scientific community associated with data science (including representatives of the Bioinformatics community) detailing an action plan to improve the connectivity speed and bandwidth required for efficient data transfer and send it as an initiative to the *Secretaría de Comunicaciones y Transportes* [Communications and Transport Secretariat] (SCT), the national entity in charge of development in the field of information and communication technologies.

Finally, a center or institute dedicated to Bioinformatics would help to consolidate a critical mass of researchers, to support regional research centers, academic institutions, and the private sector for the solution of local or national problems and collaborate to avoid effort duplication. This center or institute might host the computational infrastructure and serve as a software repository needed for the development of bioinformatics applications. This approach has been implemented successfully in other countries [5, 6, 33].

### Increase project financing in bioinformatics

Most bioinformatics projects receive financial support from public funds, mainly from the CONACYT, and a minor contribution from the private sector (Fig 2). One of the recommendations to overcome this weakness is that science and technology financing institutions in Mexico must include experts in bioinformatics into their advisory committees for the timely identification of strategic projects involving bioinformatics as a catalyst for different sectors. These committees may also suggest more efficient and more flexible financing mechanisms better adapted to technological advances. For example, in several countries, a bottleneck has been identified in the use of public resources to pay for services in the cloud, so, these committees may advise on the regulatory and operative modifications required to overcome this problem.

### Promote the maturation of regulatory frameworks for biological data management

Data of biological origin is assumed to be confidential [46–48]. This data must only be accessed after the informed knowledge of the owner, with identity protection and underwritten authorization. In Mexico, some databases contain genetic profiles for clinical research purposes, such as the *Genoteca Indígena* [Indigenous Library], providing the genetic information of people belonging to native populations [49].

Poor management of genetic data may lead to unethical behaviors. For example, an employer could identify a confidential employee health-related risk leading to an illegal job termination. In a similar way, confidential genetic information may be used for the illegal denial of medical or life insurance, as well as charging different premiums. Moreover, commercial use of personal genetic information may lead to different scenarios where particular persons or organizations could take advantage. In extreme cases, this could lead to unacceptable actions such as eugenics.

The right to non-interference in private life is recognized in the Universal Declaration of Human Rights. Unlike other countries, in Mexico the recognition of the protection of personal data in the hands of companies was only recently adopted, generating a delay in regulatory affairs. Given that this type of data has a high economic and social value, its protection is necessary to generate trust between customers and users to consolidate applications and businesses where bioinformatics intervenes [50].

Although the legal framework for the protection of personal data in possession of service companies established in Mexico includes international standards (such as those established in the conventions of the United Nations Educational, Scientific, and Cultural Organization (UNESCO) [51]) and that it has been recognized in the formulation of national laws (such as the Federal Law on the Protection of Personal Data in Possession of Individuals and the General Law on the Protection of Personal Data in Possession of Regulated Subjects [50]), the implications of the handling of information, the lack of ethics and the ignorance of the legal mechanisms to demand this right is currently a big issue in the country.

Another aspect associated with data regulation, in which the respondents showed concern, is that the mechanisms or protocols on the management of biological information, derived from projects obtained through public financing, are not clear. Expert panels could define a clear protocol for the management of this biological information in the biosciences with the inclusion of experts in Bioinformatics. This protocol should be aimed at allowing more efficient management of these information resources and facilitate the generation of research and development that result in applications and public welfare.

### Promote services and development of bioinformatics applications

It should be mentioned that biotechnology in Mexico is growing fast, with currently 406 companies that develop or use Biotechnology requiring Bioinformatics applications development. Technologies related to Bioinformatics, such as nucleic acids massive sequencing techniques, are reaching a stage of maturity and low costs favoring the development of bioinformatics applications. One idea provided by the respondents is that in the short term, Mexican researchers might participate by creating basic bioinformatics applications in order to make them friendly for the non-computer experts and, in the long term, by developing more complex and sophisticated applications.

"I believe that there is still a niche of opportunity because as there are not so many experts, the demand is growing. Then, is this niche where you can provide solutions for these groups that do not have experts, but they need to analyze data. So, the databases or the programs [to be developed] do not have to be sophisticated or innovative, but friendly for those who are not experts"

(Respondent 7).

Regarding the availability of human resources towards the development of applications, it should be mentioned that in Mexico, there are approximately 614 academic programs in areas related to Bioinformatics (Biotechnology, Biochemical Engineering, Biology, Biomedical Sciences, Pharmaceutical Chemistry and Biochemistry). Besides, more than 8,500 researchers are working in the biotechnology and life sciences areas [14]. On the side of information technologies in terms of human resources, Mexico has about 625,000 IT professionals [52]. The promotion of collaborative work between human resources from information technologies and biosciences areas would strengthen the interdisciplinary development of applications in Bioinformatics for strategic sectors such as the pharmaceutical and agri-food industries and healthcare services. In the field of the pharmaceutical industry, Mexico has been an important producer of high-tech products such as antibiotics, anti-inflammatories, antibodies, vaccines, antivenoms, chemotherapies, among other molecules, being the leading exporter of Latin America in 2015 according to Global Trade Atlas [53]. At the international level, the leading pharmaceutical companies face the challenge of patents expiration of their main products. In response, they innovate in both products and their business models, increasing their presence in emerging markets and seeking to develop innovative and specialized medicines. In this context, the application of Bioinformatics in drug development is considered of great value, since it can reduce production cost and time by 30 percent [54]. Other important developments might arise from the need for new antibiotics or orphan drugs.

In the healthcare sector, the development of Bioinformatics applications for the evaluation of health risks of individuals or populations based on their genome analysis (mainly identification of genetic or genomic risks), can help to innovate in the provision of personal and non-personal health services. For example, the timely diagnosis or identification of risk conditions (coming from heritage, lifestyle or environment) could lead to better clinical or public health decisions. About, the development of biomedical informatics as a discipline becomes increasingly important [55].

According to the need for bioinformatics applications development, it should be noted that Mexico is still in a pre-competitive state compared to other countries in the development of Bioinformatics services being most of them offered by the public academic field or by small companies with small portfolios of basic services.

"Recently there has been a proliferation of companies that provide computer consulting for bioinformatics… for basic services that are computer-based, they measure well and do it well… but when there is a problem that requires more innovation, it’s more complicated…"

(Respondent 5).

The implementation of the actions described in this paper could promote the development of more sophisticated and ambitious applications and services in Mexico to participate in the world market.

### Recommendations for a public policy

A science and technology public policy is the allocation of resources to serve the best interest of citizens. Besides, to develop public policy, it is fundamental to assess the social benefits of an investment project. The public investment in science and technology has been frequently hampered by the complexities in the identification of these benefits. In order to face this problem, it may help to identify two layers of beneficiaries: an upper one, formed by local or national governments, primary or secondary industries, and marketing industries; and a lower one, that would be composed by labor, minority, ethnic, gender, low-income or disadvantaged groups [56]. By using this differentiation, it might be easier to assess the social benefits of a scientific project aiming at the lower layer through the satisfaction of the upper one.

Also, it should be important to take the lessons learned from other countries. In this regard, the experience of the closely related Human Genome Project (HGP) may be useful to identify the best practices when proposing a national investment project in Bioinformatics. The critical route followed by the HGP started with a meeting at the University of California in Santa Cruz to discuss the feasibility of sequencing the human genome in May 1985. The academic discussion in the United States continued until mid-1986, and it is only after a high-level meeting at Cold Spring Harbor Laboratory that the Department of Energy (DOE) allocated 5.3 million dollars for the project launching stage. Additional investment from the United States and other countries was added to the project, becoming a multinational initiative. The program finished four years before, and with 10% of the predicted cost in savings, the full sequence of the human genome was published in February 2001. By that time and thanks to the input provided by 2.7 billion dollars of public funding, a new industry with a market value of 25 billion dollars was created [57], the cost of sequencing an individual human genome dropped from 100 million dollars to less than 10,000 dollars while the sequencing speed increased one million times. In brief, it was a remarkable example of a well diagnosed, planned, and deployed science public policy derived from the active involvement of the scientific community.

Another example is Genomics England, which was announced in 2013, with the aims of bringing benefit to patients, creating an ethical and transparent program based on consent, enabling new scientific discovery and medical insights and impel the development of a UK genomics industry. A year later, the program had granted £32 million directly allocated by the Wellcome Trust, the Medical Research Council, and the associated US Food and Drug Administration to individual research projects within the strategic domains. As part of the UK Industrial Strategy, the government allocated £20 million to promote the field.

In Mexico, there is little expertise on how to deploy large science strategic projects. Talking as an example of the development of Bioinformatics in the health sector, the political rise of this field is a product of its perceived value for the biomedical innovation processes. In this regard, the outline of a Mexican public policy for bioinformatics in this sector should not be located at the CONACYT level only. However, it must be the product of an agreement at the higher level involving all the public health services providers (*Secretaría de Salud* [Health Ministry] and social security institutions) along with the *Secretaría de Economía* [Ministry of Economics] and the scientific community.

## Conclusions

By consulting researchers in the field of Bioinformatics in Mexico, we present here a diagnostic of the current situation and general recommendations to accelerate the development of the area. Bioinformatics in Mexico is in a pre-competitive state, despite this, it is present in different areas of the academic sector and in a number of small companies that, together, might be the seed for the transition of national bioinformatics into a competitive state.

We propose the establishment of a network of diverse agents to plan, implement, coordinate, and evaluate strategic actions aimed to improve education and infrastructure in bioinformatics. These actions will lead to set the value of biological data generated from Mexican biodiversity.

These strategic actions, as a whole, would boost the country’s scientific and technological development, particularly in the life sciences and related economic sectors such as health care, agriculture, environment, and bio-industry (biotechnology, pharmaceutical and food industries).

In the agricultural and environmental sectors, applications based on bioinformatics stand out for the characterization of species, colonies, and ecosystems that make up the country’s biodiversity. In this area, we identified the need for a national repository of information on Mexican species and, therefore, higher public investment in this field.

In the health sector, the broad potential of bioinformatics becomes increasingly crucial for the identification and characterization of health risks, and better care practices leading to improve the health conditions of the population. As in other sectors, it is essential to seek dialogue among the multiple actors involved, including the participation of public and private entities.

In conclusion, we propose an action plan to be performed by different actors for the promotion of bioinformatics composed by several key measures: at the private sphere, coordination to promote innovation; at the public sphere, promotion of public investment in the form of a national research initiative; at the legislative sphere, the approach to the Comisión de Ciencia y Tecnología del Senado [*Science and Technology Commission of the Senate*] for the elaboration of a modern regulatory framework. To reach success, these actions should be embraced at the highest possible level of the government as a public policy.

## Supporting information

S1 File Interview guide on bioinformatics in Mexico (English–Spanish). (DOCX)

S2 File Questionnaire on bioinformatics in Mexico (English–Spanish). (DOCX)

S1 Checklist Consolidated criteria for reporting qualitative studies (COREQ) checklist. (DOCX)

S1 Dataset Additional data gotten from the questionnaire. The profile and general characteristics of the respondents are shown in Table 2.
(DOCX)
