# Supplementary material for: Bioinformatics in Mexico: A diagnostic from the academic perspective and recommendations for a public policy
Source: PLoS One. 2020 Dec 15;15(12):e0243531. doi: 10.1371/journal.pone.0243531 (PMC7737905; doi:10.1371/journal.pone.0243531)
Supplement: S1 File — (DOCX) [file pone.0243531.s001.docx]

**S1 File**

**Interview guide on bioinformatics in Mexico**

**English**

Thank you for accessing the interview,

*Explore participant profile and experience in the field of bioinformatics*

Could you please tell me about your academic background and your academic and research experience in the field of bioinformatics?

*Explore the development of bioinformatics in Mexico*

Could you comment on how you have seen the development of bioinformatics in Mexico?

*Explore the strengths and weaknesses of the current situation of bioinformatics in Mexico*

Currently, how do you think the bioinformatics situation in Mexico is?

[Explore Strengths, Weaknesses ...]

In comparison with other countries, how would you position Mexico in the field of bioinformatics?

*Explore threats to bioinformatics in Mexico*

What factors do you think could limit or hinder the development of bioinformatics in Mexico?

*Explore opportunities for bioinformatics in Mexico*

In this sense, what opportunities do you think there are for bioinformatics in Mexico?

What factors do you think could favor the development of bioinformatics in Mexico?

*Explore the development of bioinformatics in particular economic sectors*

Regarding the health issue, how do you think the bioinformatics situation in Mexico is?

What opportunities or potentialities do you think there are for bioinformatics concerning health?

[Explore: Clinical Diagnosis]

What factors do you think could favor the development of bioinformatics in this sector?

Returning a little to the question, what factors do you think might limit or hinder the development of bioinformatics in this sector?

[Explore other sectors: agricultural, environment, food, others]

*Explore other comments or perspectives of the interviewee:*

Anything you would like to comment on the subject?

**Español**

Gracias por acceder a la entrevista,

*Explorar perfil del participante y experiencia en el campo de la bioinformática*

¿Me podría platicar por favor sobre su formación académica y su experiencia académica e investigación en el campo de la bioinformática?

*Explorar sobre el desarrollo de la bioinformática en México*

¿Me podría comentar sobre cómo ha visto el desarrollo de la bioinfomática en México?

*Explorar fortalezas y debilidades sobre la situación actual de la bioinformática en México*

¿Actualmente, como cree que esté la situación de la bioinformática en México?

[Explorar Fortalezas, Debilidades…]

¿En comparación con otros países, cómo posicionaría a México en el campo de la bioinformática?

*Explorar amenazas para la bioinformática en México*

¿Qué factores cree que podrían limitar u obstaculizar el desarrollo de la bioinformática en México?

*Explorar oportunidades para la bioinformática en México*

En este sentido, ¿Qué oportunidades cree que haya para la bioinformática en México?

¿Qué factores cree que podrían favorecer el desarrollo de la bioinformática en México?

*Explorar sobre el desarrollo de la bioinformática en sectores particulares*

En relación al tema de salud, ¿Cómo cree que esté la situación de la bioinformática en México?

¿Qué oportunidades o potencialidades cree que haya para la bioinformática en relación a la salud?

[Explorar: Diagnóstico clínico]

¿Qué factores cree que podrían favorecer el desarrollo de la bioinformática en este sector?

Regresando un poco a la pregunta, ¿Qué factores cree que podrían limitar u obstaculizar el desarrollo de la bioinformática en este sector?

[Explorar otros sectores: agropecuario, medio ambiente, alimentos, otros]

*Explorar otros comentarios o perspectivas del entrevistado:*

¿Algo que le gustaría comentar en relación al tema?
