# Supplementary material for: Bioinformatics in Mexico: A diagnostic from the academic perspective and recommendations for a public policy
Source: PLoS One. 2020 Dec 15;15(12):e0243531. doi: 10.1371/journal.pone.0243531 (PMC7737905; doi:10.1371/journal.pone.0243531)
Supplement: S2 File — (DOCX) [file pone.0243531.s002.docx]

**S2 File**

**Questionnaire on Mexican Bioinformatics**

**English**

1. The type of institution to which you belong is:

   🞏 Public
   🞏 Private
2. What are your current functions in your institution?

   🞏 Academic / Researcher
   🞏 Head of Bioinformatics Services
3. What economic sector(s) does your research work focus on?

   🞏 Health
   🞏 Agricultural
   🞏 Pharmaceutical
   🞏 Environment
   🞏 Food
   🞏 Information and communication technologies
   🞏 Others
4. Could you mention what areas of research or services does your laboratory cover?

[Open answer]

1. If you have managed to develop bioinformatics tools, which ones have you developed?

[Open answer]

1. If you have successfully built bioinformatics databases, which ones have you developed?

[Open answer]

1. What software infrastructure do you have for your research and (or) services?

[Open answer]

1. Could you mention which sources are funding or have funded your research and (or) services?

   🞏 CONACYT (National Council for Science and Technology)
   🞏 State Governments
   🞏 SEP (Ministry of Public Education)

🞏 SAGARPA (Ministry of Agriculture and Rural Development)
🞏 Ministry of Health

🞏 SEMARNAT (Ministry of Environment and Natural Resources)

🞏 Other national public institutions
🞏 National Private Institutions
🞏 Private investment

🞏 Foreign institutions or agencies
🞏 Others

1. If your institution offers academic training, what opportunities do you identify to improve education in bioinformatics?

[Open answer]

1. Are you familiar with collaboration or communication networks within Mexican bioinformatics? if yes, please mention the associated network(s).

[Open answer]

1. What do you think are the main barriers to the development of bioinformatics in Mexico?

   🞏 Lack of public investment
   🞏 Lack of private investment
   🞏 Lack of specialized human resources in the field of bioinformatics
   🞏 Lack of communication between public institutions
   🞏 Lack of communication or public-private relationship
   🞏 Lack of collaboration between researchers in the field of bioinformatics
   🞏 Lack of technological infrastructure (storage and processing centers)
   🞏 Lack of regulations/normativity

**Español**

1. El tipo de institución a la que pertenece es:

🞏 Pública

🞏 Privada

1. ¿Cuáles son sus funciones actuales en su institución?

🞏 Académico / Investigador

🞏 Jefe de Servicios de Bioinformática

1. ¿En qué sectores económicos se enfoca su trabajo de investigación?

🞏 Salud

🞏 Agrícola

🞏 Farmacia

🞏 Medio ambiente

🞏 Alimentos

🞏 Tecnologías de la información y la comunicación.

🞏 Otros

1. ¿Podría mencionar que áreas de investigación o servicios abarca su laboratorio?

[Respuesta abierta]

1. En caso de haber logrado desarrollar herramientas bioinformáticas ¿cuáles ha desarrollado?

[Respuesta abierta]

1. En caso de haber logrado construir bases de datos en bioinformática ¿cuáles ha desarrollado?

[Respuesta abierta]

1. ¿Con qué infraestructura de Software cuenta para su investigación y(o) servicios?

[Respuesta abierta]

1. ¿Podría mencionar qué fuentes financian o han financiado sus proyectos de investigación en bioinformática?

🞏 CONACYT (Consejo Nacional de Ciencia y Tecnología)

🞏 Gobiernos estatales

🞏 SEP (Secretaría de Educación Pública)

🞏 SAGARPA (Secretaría de Agricultura y Desarrollo Rural)

🞏 Secretaría de salud

🞏 SEMARNAT (Secretaría de Medio Ambiente y Recursos Naturales)

🞏 Otras instituciones públicas nacionales

🞏 Instituciones privadas nacionales

🞏 Inversión privada

🞏 Instituciones u organismos extranjeros

🞏 Otros

1. En caso de que su institución ofrezca formación académica ¿Qué oportunidades identifica para mejorar la educación en materia de bioinformática?

[Respuesta abierta]

1. ¿Conoce redes de colaboración y comunicación dentro de la bioinformática mexicana? de ser afirmativo, por favor mencione la red o redes asociadas.

[Respuesta abierta]

1. ¿Cuáles cree que son las principales barreras para el desarrollo de la bioinformática en México?

🞏 Falta de inversión pública

🞏 Falta de inversión privada

🞏 Falta de recursos humanos especializados en el campo de la bioinformática.

🞏 Falta de comunicación entre las instituciones públicas.

🞏 Falta de comunicación o relaciones público-privadas

🞏 Falta de colaboración entre investigadores en el campo de la bioinformática

🞏 Falta de infraestructura tecnológica (centros de almacenamiento y procesamiento)

🞏 Falta de regulación / normatividad
