# Supplementary material for: Bioinformatics in Mexico: A diagnostic from the academic perspective and recommendations for a public policy
Source: PLoS One. 2020 Dec 15;15(12):e0243531. doi: 10.1371/journal.pone.0243531 (PMC7737905; doi:10.1371/journal.pone.0243531)
Supplement: S1 Checklist — (DOCX) [file pone.0243531.s003.docx]

**S1 Checklist**

**Consolidated criteria for reporting qualitative studies (COREQ): 32-item checklist**

| **No** | **Item** | **Guide questions/description** |
| --- | --- | --- |
| **DOMAIN 1: RESEARCH TEAM AND REFLEXIVITY** | | |
| ***Personal Characteristics*** | | |
| 1. | **Interviewer/**  **facilitator** | **Which author/s conducted the interview or focus group?**  Dagoberto Armenta and Christian Díaz de León conducted the fieldwork, including interviews and questionnaires. |
| 2. | **Credentials** | **What were the researcher's credentials? *e.g. PhD, MD***  Dagoberto Armenta: PhD (Biotechnology, bioinformatics)  Christian Díaz de León: PhD (Public Health, Health systems)  Brenda Valderrama: PhD (Biotechnology) |
| 3. | **Occupation** | **What was their occupation at the time of the study?**  Dagoberto Armenta: Academic and Researcher in Bioinformatics  Christian Díaz de León: Academic and Researcher in eHealth, digital health  Brenda Valderrama: Academic and Researcher in Biotechnology |
| 4. | **Gender** | **Was the researcher male or female?**  Dagoberto Armenta: Male  Christian Díaz de León: Male  Brenda Valderrama: Female |
| 5. | **Experience and training** | **What experience or training did the researcher have?**  Dagoberto Armenta: Biotechnology and Bioinformatics  Christian Díaz de León: eHealth, digital health, Qualitative research, Public health policy  Brenda Valderrama: Biotechnology and bioinformatics, Science and technology policy |
| ***Relationship with participants*** | | |
| 6. | **Relationship established** | **Was a relationship established prior to study commencement?**  No, only some of the participants were known previously principally by Dagoberto Armenta, due to his academic experience in the bioinformatics field in Mexico. |
| 7. | **Participant knowledge of the interviewer** | **What did the participants know about the researcher? e*.g., personal goals, reasons for doing the research***  The participants were informed about the study proposals and objectives, the researchers' names, occupations, and affiliations. |
| 8. | **Interviewer characteristics** | **What characteristics were reported about the interviewer/facilitator? e.g. *Bias, assumptions, reasons and interests in the research topic***  Along with the objectives and proposals of this research, the reasons and interests were informed to the participants. The main bias of this study could be that the first and third researches are involved in this field of study (bioinformatics). |
| **DOMAIN 2: STUDY DESIGN** | | |
| ***Theoretical framework*** | | |
| 9. | **Methodological orientation and Theory** | **What methodological orientation was stated to underpin the study? *e.g. grounded theory, discourse analysis, ethnography, phenomenology, content analysis***  The major methodological orientation was Phenomenology. Besides, a quantitative technique was incorporated, which is an online survey applied to the same participants of the study. |
| ***Participant selection*** | | |
| 10. | **Sampling** | **How were participants selected? *e.g. purposive, convenience, consecutive, snowball***  Several sampling techniques were used, such as purposive, convenience, and snowball. Some key respondents were identified using the information gotten from a bibliometric analysis of bioinformatics in Mexico. |
| 11. | **Method of approach** | **How were participants approached? e*.g. face-to-face, telephone, mail, email***  The participants were approached face-to-face, principally for semi-structured interviews (a telephone call scheduled only one interview). The questionnaire was applied online. |
| 12. | **Sample size** | **How many participants were in the study?**  We included a total of 26 participants, 14 of them were interviewed. |
| 13. | **Non-participation** | **How many people refused to participate or dropped out? Reasons?**  Only 1. We didn't get a response from him via email. |
| ***Setting*** | | |
| 14. | **Setting of data collection** | **Where was the data collected? e*.g. home, clinic, workplace***  Interviews were conducted in a private setting. Considering some academic events that were organized, private rooms were used. Some interviews were also performed at the office of the participants. |
| 15. | **Presence of non-participants** | **Was anyone else present besides the participants and researchers?**  No |
| 16. | **Description of sample** | **What are the important characteristics of the sample? *e.g. demographic data, date***  Being an academic or researcher in the field of bioinformatics in Mexico was the principal characteristic of the participants included in the sample. |
| ***Data collection*** | | |
| 17. | **Interview guide** | **Were questions, prompts, guides provided by the authors? Was it pilot tested?**  Yes, we designed an interview guide before the fieldwork. The pilot technique was not used; however, we adapted the interview guide during the fieldwork, considering the responses of the participants and the study objectives and interests. |
| 18. | **Repeat interviews** | **Were repeat interviews carried out? If yes, how many?**  No, any of the interviews was repeated. |
| 19. | **Audio/visual recording** | **Did the research use audio or visual recording to collect the data?**  Audio-recording was used to collect data. |
| 20. | **Field notes** | **Were field notes made during and/or after the interview or focus group?**  Field notes were taken after the interviews principally. |
| 21. | **Duration** | **What was the duration of the interviews or focus group?**  The total duration of the 14 interviews was 366 min (6h, 6 min) |
| 22. | **Data saturation** | **Was data saturation discussed?**  We looked for data triangulation to confirm the findings. Data saturation was also gotten from some of the findings, although the sample was small but representative. |
| 23. | **Transcripts returned** | **Were transcripts returned to participants for comment and/or correction?**  No, it was considered not necessary. |
| **DOMAIN 3: ANALYSIS AND FINDINGS** | | |
| ***Data analysis*** | | |
| 24. | **Number of data coders** | **How many data coders coded the data?**  Only two persons coded the data: Dagoberto Armenta and Christian Díaz de León |
| 25. | **Description of the coding tree** | **Did the authors provide a description of the coding tree?**  The principal categories were taken from the SWOT analysis (the strengths, weaknesses, opportunities, and threats). We analyzed the transcriptions to identify the subcategories inside these categories. We provide a summary of these categories and subcategories in Table 3. |
| 26. | **Derivation of themes** | **Were themes identified in advance or derived from the data?**  Themes and categories were identified from the data. |
| 27. | **Software** | **What software, if applicable, was used to manage the data?**  We used a Google Sheets shared file. Though a table built in this file, the principal identified categories and subcategories were listed, and the corresponding memos and verbatim quotes were introduced. This technique allowed analytic triangulation. |
| 28. | **Participant checking** | **Did participants provide feedback on the findings?**  The participants principally gave feedback during the interview process, looking for data triangulation (previous findings identified with other interviews). |
| ***Reporting*** | | |
| 29. | **Quotations presented** | **Were participant quotations presented to illustrate the themes/findings? Was each quotation identified? e*.g. participant number***  Yes, quotations and their respective identifications are presented (results section). |
| 30. | **Data and findings consistent** | **Was there consistency between the data presented and the findings?**  The principal findings were illustrated with data such as quotations and pie graphs. |
| 31. | **Clarity of major themes** | **Were major themes clearly presented in the findings?**  Major themes were presented in the results section. |
| 32. | **Clarity of minor themes** | **Is there a description of diverse cases or discussion of minor themes?**  Minor themes were included in the discussion section. |
