# Supplementary material for: Bioinformatics in Mexico: A diagnostic from the academic perspective and recommendations for a public policy
Source: PLoS One. 2020 Dec 15;15(12):e0243531. doi: 10.1371/journal.pone.0243531 (PMC7737905; doi:10.1371/journal.pone.0243531)
Supplement: S1 Dataset — The profile and general characteristics of the respondents are shown in Table 2. (DOCX) [file pone.0243531.s004.docx]

**S1 Dataset. Adittional data gotten from the questionnaire^a^**

| **Respondent Number** | **What economic sector (s) does your research work focus on?** | **Could you mention what areas of research or services does your laboratory cover?** | **If you have managed to develop bioinformatics tools, which ones have you developed?** | **If you have successfully built bioinformatics databases, which ones have you developed?** | **What software infrastructure do you have for your research and (or) services?** | **Could you mention which sources are funding or have funded your bioinformatics research projects?** | **If your institution offers academic training, what opportunities do you identify to improve education in bioinformatics?** | **Are you familiar with collaboration or communication networks within Mexican bioinformatics? if yes, please mention the associated network(s).** | **What do you think are the main barriers to the development of bioinformatics in Mexico?** |
| --- | --- | --- | --- | --- | --- | --- | --- | --- | --- |
| 1 | Health,  Agricultural,  Basic Science (although it is not an economic sector) | Bioinformatics, Genomics, Computational Biology | TargetExpress |  | Free software: Linux and diverse packages | Conacyt,  SEP,  Foreign institutions or agencies | Improvements at high-shool and/or bachelor's levels. | A bioinformatics collaboration network is beginning | Lack of public investment,  Lack of private investment,  Lack of specialized human resources in the field of bioinformatics,  Lack of communication between public institutions,  Lack of communication or public-private relations,  Lack of collaboration between researchers in the field,  Lack of infrastructure technological (storage and processing centers) |
| 8 | Health | General Cellular and Molecular Biology. General Bioinformatics. | SurvExpress, SurvMicro, VALORATE, PubTerm,  GALGO | SurvExpress, SurvMicro | Free software | Conacyt,  National private institutions | That the students of the life sciences area take computer science and bioinformatics subjects and that computer science students take biology and bioinformatics subjects. | I don't know.  I think you should create one. | Lack of specialized human resources in the field of bioinformatics,  Lack of collaboration between researchers in the field,  Lack of technological infrastructure (storage and processing centers) |
| 10 | Health,  Agricultural, Environment, Pharmaceutical, Food,  Information and Communication Technologies | Basic sciences, Agriculture,  Veterinary and human health. | Python, perl, bash,  R scripts focused on automation and specialized analysis | Viral, bacterial and plants. | Open-source focused on OMICS | Conacyt,  Sagarpa,  Secretaría de Salud,  State Governments,  Other national public institutions,  National private institutions,  Foreign institutions or agencies | Overseas training focused on OMICS. | I was a Bioinformatics student in Argentina.  Latin American Bioconductor Network. | Lack of public investment,  Lack of private investment,  Lack of specialized human resources in the field of bioinformatics,  Lack of communication between public institutions,  Lack of communication or public-private relations,  Lack of collaboration between researchers in the field,  Lack of infrastructure technology (storage and processing centers),  Lack of regulations |
| 11 | Health | Bioinformatics support for big data analysis provided | - | - |  | Other national public institutions | There is a great need to learn the basic knowledge of bioinformatics, to understand the results of the analyzes | Only the bioinformatics node hosted by UNAM | Lack of specialized human resources in the field of bioinformatics,  Lack of technological infrastructure (storage and processing centers),  Lack of regulations |
| 13 | Health,  Agricultural, Environment, Pharmaceutical | We perform simulations by molecular dynamics, small and large scale molecular coupling (molecular screening), and modeling of proteins and complexes. | none | none | NAMD, CHARMM, and online servers. | Conacyt | Currently, we have an area of ​​specialization in the Postgraduate in Sciences, represented by researchers who do bioinformatics. If it matures, a well-defined area can be made. | The REFEP (Protein Structure, Function and Evolution Network) | Lack of public investment,  Lack of private investment,  Lack of specialized human resources in the field of bioinformatics,  Lack of communication or public-private relations,  Lack of technological infrastructure (storage and processing centers) |
| 15 | Health, Environment | Genomics and transcriptomics of model or non-model organisms. |  |  | Many different modules in the cluster (too many to name in this space) | Conacyt,  Foreign institutions or agencies | The development of learning programs with a practical focus and in Spanish. | Yes | Lack of private investment,  Lack of specialized human resources in the field of bioinformatics,  Lack of communication between public institutions,  Lack of communication or public-private relations,  Lack of collaboration between researchers in the field |
| 16 | Health,  Agricultural, Environment,  Food | Genomic diagnosis |  |  | Derivatives of scientific and open source articles | Conacyt,  State Governments,  Other national public institutions | This area needs a strong background in many disciplines | Yes | Lack of specialized human resources in the field of bioinformatics,  Lack of technological infrastructure (storage and processing centers) |
| 17 | Health,  Agricultural | Omic data analysis |  |  | Many open access programs | Other national public institutions |  | No | Lack of public investment,  Lack of specialized human resources in the field of bioinformatics,  Lack of communication between public institutions,  Lack of technological infrastructure (storage and processing centers) |
| 18 | Agricultural, Environment, Information and Communication Technologies | Bioinformatics,  Genetic engineering, Bioprocesses,  Natural products,  Agro-industrial waste | Regulation networks in FPGAs,  Algorithm to find metabolic routes | Eragen;  Databases on metabolic pathways | Own development | Indirectly by technological innovation funds | BigData, FPGAs | I don't know | Lack of public investment,  Lack of private investment,  Lack of specialized human resources in the field of bioinformatics,  Lack of communication between public institutions,  Lack of collaboration between researchers in the field,  Lack of technological infrastructure (storage and processing centers) |
| 19 | Health,  Food,  Information and Communication Technologies | Bioinformatics,  New Generation Sequencing and Genomics,  Molecular Modeling. | Software for the design of probes for microarrays.  Virtual Hybridization Analysis.  VAMPhyRE: Software for comparative genomics analysis | We have not developed databases. | Our support in Software infrastructure has so far been based on Free Software projects specialized in Bioinformatics. All our equipment is based on Linux and Unix / Apple. There are licenses for Embarcadero Delphi and Active State programming software. | Conacyt,  Other national public institutions | Our Institution offers since 2002 basic bioinformatics courses at the postgraduate level, focused on researchers in the biological area. Starting this year, we also offer an advanced bioinformatics course for processing large volumes of data. At the same time, there are currently modifications in the study plans of the bachelor's degree programs offered in our school to include bioinformatics as a basic or complementary course in academic training. | Yes, particularly The National Bioinformatics node of UNAM. | Lack of public investment,  Lack of private investment,  Lack of specialized human resources in the field of bioinformatics,  Lack of communication between public institutions,  Lack of communication or public-private relations,  Lack of collaboration between researchers in the field,  Lack of infrastructure technology (storage and processing centers),  Lack of regulations |
| 20 | Health, Environment | Evolutionary genomics of pathogenic bacteria |  | Local databases for our projects | Different free programs | Conacyt,  UNAM | The teaching of specialized courses | No | Lack of public investment,  Lack of private investment,  Lack of specialized human resources in the field of bioinformatics,  Lack of communication between public institutions,  Lack of communication or public-private relations |
| 21 | Health, Environment, Pharmaceutical | Molecular modeling in biological systems  Development of nano-biosensors | Drug search process with cancer applications |  | Molecular Dynamics, Quantum mechanics, QM / MM, Molecular Docking, etc. | Conacyt,  Other national public institutions,  Foreign institutions or agencies,  National and international collaborations | They need to learn Linux, programming and basic computer tools | No | Lack of private investment,  Lack of specialized human resources in the field of bioinformatics,  Lack of collaboration between researchers in the field,  Lack of technological infrastructure (storage and processing centers) |
| 22 | Health, Pharmaceutical, Information and Communication Technologies | Identification of antimicrobial peptides, drug design, identification of heat-sensitive mutants, identification of transcription factor binding sites in cancer |  |  |  | Conacyt,  Other national public institutions | Generation of a critical mass of specialists | Thematic Network of Proteins, Prions, and Neurodegenerative Diseases.  Thematic Network of Structure, function, and evolution of proteins. | Lack of public investment,  Lack of private investment,  Lack of specialized human resources in the field of bioinformatics,  Lack of communication between public institutions,  Lack of communication or public-private relations |
| 23 | Health,  Pharmaceutical,  Genomic services | Disease Risk, Pharmacogenomics and Ancestry | Automation of SNP analysis to generate a report on risk, Pharmacogenomics and Ancestry. | Database with genetic information specific to the Mexican population. | Illumina Software, BC Platforms and in-house development. | Conacyt,  Private investment | Social service | No | Lack of public investment,  Lack of private investment,  Lack of specialized human resources in the field of bioinformatics,  Lack of communication between public institutions,  Lack of communication or public-private relations,  Lack of collaboration between researchers in the field,  Lack of infrastructure technology (storage and processing centers),  Lack of regulations |
| 24 | Health, Environment, Pharmaceutical |  |  |  |  | Foreign institutions or agencies |  | No | Lack of public investment,  Lack of private investment,  Lack of specialized human resources in the field of bioinformatics,  Lack of communication between public institutions,  Lack of communication or public-private relations,  Lack of collaboration between researchers in the field |
| 25 | Pharmaceutical | Medicinal chemistry, bioinformatics, molecular modeling | None | None | Free online programs, and others installed autodock, namd, gromacs, and purchase amber and gaussian. | Conacyt,  State Governments | We offer courses each semester to the students of our postgraduate courses and pay to external students. | No | Lack of specialized human resources in the field of bioinformatics,  Lack of communication between public institutions,  Lack of collaboration between researchers in the field |
| 26 | Health,  Agricultural | Protein engineering, sequence analysis, bioinformatics | HIP system for comparison of sequence entropy profiles for identification of remote homologs. |  | Lots of free software | Conacyt,  Secretaría de Salud,  Other national public institutions | Professionalization of the curriculum | No | Lack of public investment,  Lack of private investment,  Lack of specialized human resources in the field of bioinformatics |

^a^The characteristics of the respontents are shown in the Table 2 of the manuscript.
